# An Intensive Culinary Intervention Programme to Promote Healthy Ageing: The SUKALMENA-InAge Feasibility Pilot Study

**DOI:** 10.3390/nu16111735

**Published:** 2024-06-01

**Authors:** Jara Domper, Lucía Gayoso, Leticia Goni, Laura Perezábad, Cristina Razquin, Victor de la O, Usune Etxeberria, Miguel Ruiz-Canela

**Affiliations:** 1 Basque Culinary Center, Faculty of Gastronomic Sciences, Mondragon Unibertsitatea, 20009 San Sebastián, Spain; jdomper@bculinary.com (J.D.); lgayosom@gmail.com (L.G.); lperezabad@bculinary.com (L.P.); uetxeberria@bculinary.com (U.E.); 2 BCC Innovation, Technology Center in Gastronomy, Basque Culinary Center, 20009 San Sebastián, Spain; 3 Department of Preventive Medicine and Public Health, Instituto de Investigación Sanitaria de Navarra (IdiSNA), University of Navarra, 31008 Pamplona, Spain; lgoni@unav.es (L.G.); crazquin@unav.es (C.R.); victor.delao@unir.net (V.d.l.O.); 4 Consorcio Centro de Investigaciones Biomédicas en Red (CIBEROBN), Institute of Health Carlos III (ISCIII), 28029 Madrid, Spain; 5 Faculty of Health Sciences, International University of La Rioja (UNIR), 26006 Logroño, Spain

**Keywords:** home food preparation, home cooking, gastronomy, culinary medicine, cooking skills, health promotion, obesity/overweight, cooking classes, non-communicable chronic diseases

## Abstract

Dietary interventions are a key strategy to promote healthy ageing. Cooking skills training emerges as a promising approach to acquiring and maintaining healthy eating habits. The purpose was to evaluate the effectiveness of a culinary programme to improve healthy eating habits among overweight/obese adults (55–70 years old). A total of 62 volunteers were randomly (1:1) assigned to an culinary intervention group (CIG) or a nutritional intervention group (NIG). Dietary, cooking, and health-related outcomes, including body advanced glycation end product (AGE) levels, were evaluated at baseline and after four weeks. Mixed-effects linear models were used to assess the effects of the interventions within and between groups. Among the 56 participants who completed the trial, CIG participants achieved a significant improvement in Mediterranean diet adherence (1.2; 95%CI, 0.2 to 2.2) and a reduction in the use of culinary techniques associated with a higher AGE formation in foods (−2.8; 95%CI, −5.6 to −0.2), weight (−1.5; 95%CI, −2.5 to −0.5), body mass index (−0.5; 95%CI, −0.8 to −0.2), waist circumference (−1.4; 95%CI, −2.6 to −0.2), and hip circumference (−1.4; 95%CI, −2.4 to −0.4) compared with the NIG participants. Although a greater confidence in cooking in the CIG was found, attitudes and cooking habits did not improve. No significant differences in biochemical parameters or AGEs were found between groups. In conclusion, a culinary intervention could be successful in promoting healthy eating and cooking habits compared to a programme based on nutrition education alone. Nevertheless, further efforts are needed to strengthen attitudes and beliefs about home cooking, to address potential barriers and understand the impact of cooking interventions on biological parameters. Larger studies with longer follow-ups are needed to evaluate the relationship between cooking, diet, and health.

## 1. Introduction

Ageing, a natural life process, has been linked to the onset of non-communicable chronic diseases (NCDs). Both ageing and NCDs share certain biological processes, such as the exacerbation of inflammation process or oxidative stress [1]. NCDs have become the leading cause of disability during ageing, and this factor can lead to declining health in old age [1], emphasizing the need to reduce the risk of NCDs and their comorbidities to promote healthy ageing [2]. One important risk factor of NCDs is overweight and obesity [3].

Healthy ageing is defined as “the process of developing and maintaining the functional ability that enables well-being in older adults” [4]. The development of preventive interventions to reduce the incidence and impact of NCDs is a key aspect for the promotion of healthy ageing [1,3]. In this context, interventions related to diet and food habits are considered the main strategies for healthy ageing [2]. However, adopting and maintaining healthy eating habits remain the main barriers [5].

Training in cooking skills has emerged as a promising educational strategy in nutritional interventions, enabling the maintenance of healthy eating habits [5]. Concepts such as culinary medicine and teaching kitchens recognize cooking knowledge and skills as key resources for establishing and maintaining a healthy diet, as well as for enjoying delicious food [6,7]. Culinary medicine also seems to be a positive health strategy [6] during the ageing process [8], through dietary patterns, such as the Mediterranean diet (MedDiet) [9] or plant-based diets [10].

Moreover, beyond recommended dietary choices, the way we cook has been shown to affect health [11]. Advanced glycation end products (AGEs) are a group of heterogeneous molecules formed by the chemical reaction between sugars and proteins. These molecules are formed endogenously or can be taken from exogenous sources, including the diet [12]. Indeed, the main sources of AGEs in the body are unhealthy diets based on the high consumption of ultra-processed foods and the use of cooking techniques, which are responsible for the formation of new AGEs in food [13,14]. The accumulation of AGEs in the body has been associated with the induction of oxidative stress and inflammation and consequently the development of obesity and NCDs [12,15]. In this sense, promoting healthy eating habits and using healthier cooking techniques have been found to reduce dietary AGEs and thus their accumulation in the human body [16].

In the SUKALMENA study, we showed that a culinary intervention improved confidence in cooking at home and increased the use of healthy culinary practices in people with type 2 diabetes compared to a nutritional intervention [17]. However, more efforts are needed to measure the effect of culinary interventions on the health of ageing people, for example studying the impact of this type of intervention on the accumulation of body AGEs.

The SUKALMENA-InAge study is a short-term randomized controlled trial which aims to evaluate the effectiveness of a culinary intervention programme to improve nutritional and health-related outcomes among overweight and obese adults (55–70 years old).

## 2. Materials and Methods

### 2.1. Study Design

SUKALMENA-InAge is a randomized controlled trial conducted to evaluate the effectiveness of a culinary intervention on the health of middle-aged and older adults and to compare these results with the outcomes obtained from a more traditional nutrition education programme. The latter was predicated on theoretical advice from the MedDiet, whereas the culinary intervention also included training in healthy cooking. Both interventions were implemented for four weeks. During this time, two visits were planned and carried out by a dietician and a nurse: before (T1) and immediately after the end of the intervention (T2).

The protocol of the study was approved by the Drug Research Ethics Committee of the Basque Country (PI2021067), and it was conducted in compliance with the Declaration of Helsinki. Information about this study was described and given to the participants, after which the signed a written informed consent form. The trial was registered at ClinicalTrial.gov as NCT04908163.

### 2.2. Sample Recruitment and Selection

As SUKALMENA-InAge is a feasibility study, sample size calculation was not applied [18], but we estimated around 50 participants to observe intra-subject differences to be found between baseline and follow-up, similar to previous published studies [19,20]. Participants were recruited through the social networks of Basque Culinary Center (BCC) (San Sebastián, Spain) and through local mass media campaigns. The inclusion criteria were as follows: (1) age between 55 and 70 years old; (2) overweight or obesity (body mass index [BMI] between ≥25 and ≤35 kg/m^2^); and (3) a low level of culinary skills. The level of culinary skills was measured by the Home cooking Quality Index, a non-validated 19-item questionnaire (Supplementary Table S1) developed by the University of Navarra and BCC Innovation. A low level of culinary skills was considered with a score lower than 10 points. The exclusion criteria were as follows: (1) any illness that reduces the average lifespan to less than a year; (2) chronic alcoholism, the consumption of illegal drugs or alcohol > 80 g/day; (3) involvement in a medication clinical trial or diet plan the year prior to enrolment; (4) any major difficulties or inconveniences in making changes to healthy dietary habits and adhering to the MedDiet (food allergies, intolerances); (5) low motivation to change dietary habits; and (6) any medical intervention (surgical or pharmacological) or dietary plan followed to lose weight in the year prior to inclusion.

**Figure 1:**
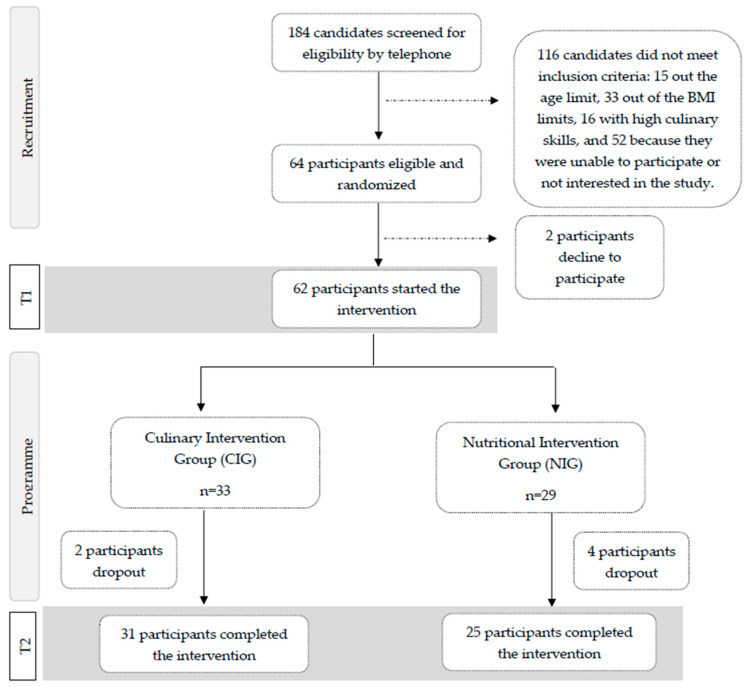
Participants’ flowchart for the SUKALMENA-InAge trial.

Of the 184 candidates interested in the study, 64 adults met all inclusion criteria after a screening interview, and 62 participants started the intervention. After signing the informed consent form, participants were randomly assigned (1:1) to one of the two intervention groups (Figure 1).

A researcher uninvolved in recruitment performed an unrestricted randomization to ensure adequate allocation concealment.

### 2.3. Intervention and Control Conditions

Of the 62 participants enrolled, 29 participants were assigned to the nutritional intervention group (NIG), and 33 were assigned to the culinary intervention group (CIG).

Participants in the CIG received a 4-week intervention programme with 2 weekly sessions. The sessions lasted 60 min and were delivered online. The first weekly session contained theoretical nutritional information and was led by a registered dietician; this session consisted of two parts, gastronomic culture and culinary resources, and included nutritional knowledge. The second session of the week consisted of cooking demonstrations led by a chef from BCC Innovation, the Technology Center specialized in Gastronomy from BCC. These demonstrations included the practical preparation of dishes by using specific culinary techniques and healthy ingredients. In addition, during the second week of the intervention, CIG participants received individual telephone coaching from dietitians. The calls lasted between 10 and 30 min and were conducted according to a protocol developed for this study.

The curriculum of the programme implemented in the CIG is summarized in Table 1.

At the start of the intervention, printed material with nutritional information was given to CIG participants to promote the MedDiet and so was access to an additional culinary resources website (49 video recipes); each week, some activities were proposed to cook recipes, and all recorded lessons were uploaded (https://sukalmena.wixsite.com/sukalmena, accessed on 31 May 2024). A book with 28 recipes was also given to them which was designed according to the dimensions of the healthy eating plate and included ideas for healthy breakfast and snacks and two weeks’ worth of menu ideas and a grocery list. In addition, each week, emails were sent to the participants with an overview of the week’s key takeaways. The culinary intervention curriculum offered not only nutritional tools, such as meal planning, but also culinary education tools to improve, among other things, the consumption of plant-based foods and healthy cooking techniques to reduce the formation of AGEs. An example of a culinary education tool could be the gastro-healthy eating plate, which includes both food groups and healthy culinary techniques that could be used to cook foods from each food group.

Materials with nutritional information on how to follow the MedDiet were handed out to the participants in the NIG. This information describes the typical MedDiet foods, the suggested amount of food per day for every food category, the healthy eating plate, advice on healthy shopping, and general information to promote a healthy lifestyle. Having completed the study, this group received the additional information provided to the CIG.

### 2.4. Outcomes Assessed

All outcomes were assessed at baseline (T1) and after the intervention (T2) in both groups. The evaluation was focused on both changes between baseline and post-intervention within the groups and changes between the two different conditions after intervention.

#### 2.4.1. Primary Outcome

The primary outcome was the change in adherence to the MedDiet measured by the 14-item Mediterranean Diet Adherence Screener (MEDAS) questionnaire. The MEDAS score can range from 0 to 14, with higher scores indicating a better adherence [21].

#### 2.4.2. Secondary Outcomes

Secondary outcomes were classified as dietary outcomes, health-related outcomes, AGE body accumulation, or cooking-related outcomes.

- Dietary outcomes

Dietary outcomes included changes in food consumption. A 143-item semiquantitative Food Frequency Questionnaire (FFQ), validated for the Spanish population [22,23], was used to examine food consumption at T1 and T2. Each question in the questionnaire had 9 options for the frequency of food consumption, ranging from never or less than once a month to more than 6 times a day. The energy and nutrient intakes were also calculated on the basis of Spanish food composition tables [24,25].

- Cooking-related outcomes

Cooking-related outcomes were measured as changes in cooking habits, confidence, and attitudes towards cooking. At T1 and T2, cooking behaviours were evaluated with a validated Home Cooking Frequency Questionnaire (HCFQ) [26]. Culinary techniques were also classified into those associated with a higher formation of AGEs (pan frying, grilling (barbecue), roasting/broiling, deep frying, and breading) and those techniques associated with lower AGE formation (steaming, stewing, boiling, microwaving, and sweating) [27].

At T1 and T2, participants completed a questionnaire adapted from Condrasky et al. (2011) [28] and Vrhovnik (2012) [29] to assess confidence and attitudes towards cooking at home. A 10-point Likert scale was applied in both situations. The response options for confidence varied from 1 (meaning “not very sure”) to 10 (meaning “very sure”). The response options for the attitude scale went from 1 (completely disagree) to 10 (absolutely agree).

- Health and biological outcomes

Health-related outcomes comprised changes in anthropometry, blood pressure, and biochemical parameters, including body AGE accumulation.

At T1 and T2, body composition and anthropometric information were collected. Tanita bioelectrical impedance (MC-780 MA, Tanita Corp, Tokyo, Japan) was used to assess the body weight and body composition. Height was measured to the nearest 1 mm using a portable stadiometer. Measurements were taken in light clothing and without shoes, by a trained nutritionist. To calculate BMI, weight (in kilograms) was divided by height (in metres squared). Waist and hip circumference were measured with a flexible tape to an accuracy of 1 mm. To assess the waist circumference, the midpoint between the border of the iliac crest and the last rib was measured, and to assess the hip circumference, the maximum circumference of the buttocks was measured. Following a 5 min rest interval, the participant was seated, and the validated automatic oscillometer (Omron M2 HEM-7102-E, The Netherlands) was used to measure the systolic (SBP) and diastolic (DBP) values in triplicate in each arm at T1 and T2.

Biochemical determinations were performed on fasting blood samples collected at T1 and T2. Serum tubes (8.5 mL) were gathered, and aliquots were coded, kept at −80 °C until analysis, and stored. Standard automated enzymatic methods were used to assess serum glucose, triglycerides, total cholesterol, high-density lipoprotein cholesterol (HDL-c), and low-density lipoprotein cholesterol (LDL-c). C-reactive protein (CRP) and tumour necrosis factor-alpha (TNF-α) were measured by an immune turbidimetric assay. Insulin was measured by a chemiluminescence immunoassay. The homeostatic model assessment of insulin resistance (HOMA-IR) was calculated according to the following equation: insulin (mcU/L) ×glucose (mg/dL)/405 [30].

AGEs were measured by collecting blood and urine samples and measuring skin autofluorescence at T1 and T2. ELISA was used to measure *N*-carboxymethyl-lysine (CML) and the receptor (extracellular domain) for AGEs (RAGE) in duplicate serum samples from every individual at every time point. Using commercial kits (Quantikine DRG00, R & D Systems, Oxford, UK, and Immunotag Human CML ELISA Kit, GBiosciences, MO, USA, respectively), the extracellular domains of RAGE (RAGE) and CML (AGE) were assessed in accordance with the manufacturer’s instructions. CML was also determined by ELISA in urine samples following the same procedure and using the same commercial kits. AGE accumulation in the skin was estimated using the AGE reader Mu Connect (Microcaya, Bilbao-Spain). This non-invasive and validated method can estimate the level of AGE accumulation in the skin by measuring the characteristic fluorescence of AGEs [31,32]. Skin autofluorescence (SAF) was measured on the volar side of the forearm on the dominant side, approximately 10 cm below the elbow. The emitted and reflected light intensities were measured with a spectrometer in the range of 300–600 nm. SAF was based on the ratio of the average intensity of the emitted light (420–600 nm) divided by the average intensity of reflected light (300–420 nm), multiplied by 100 and expressed in arbitrary units (AU). The average of three measurements was taken.

### 2.5. Other Measurements

Information on sociodemographic variables (civil status, education level, sex, and occupation) and smoking habits was collected from all participants at the baseline visit.

### 2.6. Statistical Analysis

The statistical analyses were conducted on participants with available data from visit 1 and 2. Mean ± Standard Deviation (SD) were used to describe quantitative and categorical variables. Categorical variables were expressed as percentages (%). Baseline differences in sociodemographic, dietary, and cooking characteristics between the intervention groups were analysed using Student’s *t*-test or the Mann–Whitney test for continuous variables and the chi-square test or Fisher’s exact test for categorical variables. Mixed-effects linear models were used to assess the effects of the interventions within each group, as well as differences in post-intervention changes between groups, controlling for baseline scores. Mean changes and 95% confidence intervals (95% CIs) were calculated for changes within groups and differences in changes between groups. All analyses were conducted with STATA software (STATA version 14.0, StataCorp, College Station, TX, USA), and all *p*-values presented are two-tailed. *p* < 0.05 level was considered as statistically significant.

## 3. Results

A total of 62 participants began the intervention; 29 were in the NIG, and 33 were in the CIG. After four intervention weeks, 56 participants completed the trial, 31 in the CIG and 25 in the NIG (Figure 1).

Baseline sociodemographic characteristics and smoking habits for both groups are summarized in Supplementary Table S2. At baseline, there were no significant differences between the intervention groups.

### 3.1. Dietary Outcomes

Baseline characteristics according to the intervention groups are shown in Table S2. Significant differences were observed between the NIG and CIG in the baseline consumption of legumes and *sofrito* (Table S2).

**Figure 2:**
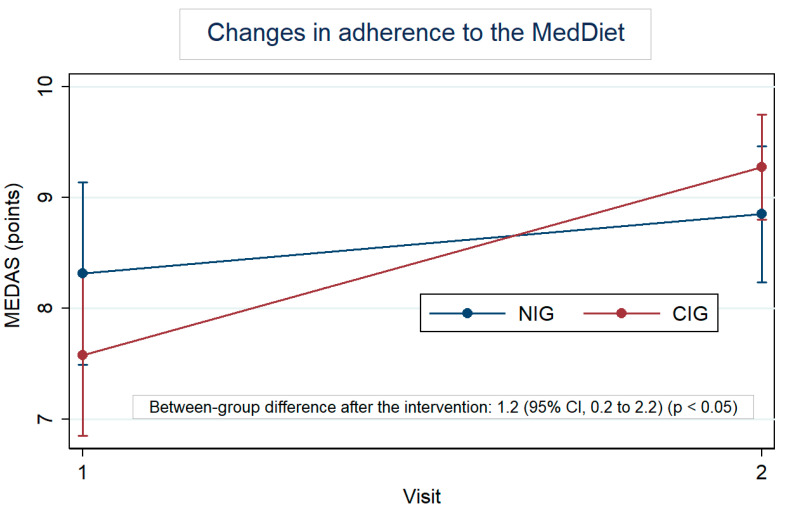
Within-group changes in the Mediterranean Diet Adherence Score (MEDAS) after the intervention adjusted for baseline values.

Figure 2 highlights the MedDiet adherence by group, at baseline and after the intervention. At baseline, the MEDAS was 8.3 for the NIG and 7.6 for the CIG. In both groups, this score increased; however, the within-change was larger in the CIG, with an increase of 1.7 points (95% CI: 1.0 to 2.4) vs. an increase of 0.5 points (95% CI: −0.2 to 1.3) in the NIG. Furthermore, the difference in change between groups was 1.2 (95% CI: 0.2 to 2.2). Table S3 also shows the changes, in percentage, of each item of the MEDAS. Regarding the results for each question, participants in the CIG significantly increased the consumption of vegetables (*p* < 0.01), legumes (*p* < 0.001), and nuts (*p* < 0.05) once they reduced the consumption of unhealthy fats (*p* < 0.05) and sweet beverages (*p* < 0.05). Lean meat consumption was also preferred over red meat by CIG participants (*p* < 0.05). Significant between-group differences were observed in the consumption of legumes (*p* < 0.001) after the intervention.

Supplementary Table S4 highlights the effect of the intervention in energy and nutrient intake. NIG participants reduced total energy intake (*p* < 0.05), animal protein consumption (*p* < 0.05), and sodium intake (*p* < 0.001) while increasing the proportion of the fat intake from monounsaturated fatty acids (MUFAs) (*p* < 0.05). CIG participants reduced the carbohydrate intake (*p* < 0.001), glycaemic load (*p* < 0.01), and glycaemic index (*p* < 0.01) of the foods consumed, animal protein intake (*p* < 0.01), and sodium intake (*p* < 0.05), while they increased total fat intake (*p* < 0.001) and fat intake from MUFAs (*p* < 0.001) and polyunsaturated fatty acids (PUFAs) (*p* < 0.01). Statistically significant between-group differences were observed with a higher reduction in carbohydrate intake (*p* < 0.05) and glycaemic index (*p* < 0.05) in the CIG compared to NIG participants, whereas a higher increase in PUFAs (*p* < 0.05) was observed in the CIG compared to NIG participants.

Regarding the effect of the intervention on food consumption (Supplementary Table S5), only a slightly higher increase was observed in the consumption of olive oil in NIG compared to CIG participants.

### 3.2. Cooking-Related Outcomes

No statistically significant differences were observed between the groups in terms of the overall confidence in cooking (Table 2), although participants in the CIG significantly increased their overall confidence in cooking after the intervention (*p* < 0.01), as well as in their confidence in preserving food (*p* < 0.05), preparing a balanced meal (*p* < 0.01), comparing prices to save money (*p* < 0.05), planning meals for the week (*p* < 0.01), modifying a recipe when they did not have an ingredient (*p* < 0.05), modifying recipes to make them healthier (*p* < 0.01), and using leftovers to create another meal (*p* < 0.05).

No significant differences were found in terms of the overall attitude about cooking at home between groups (Table 3). However, slight changes were observed in both groups at the end of the intervention. Participants in the NIG reported a decrease in their attitude towards cooking new recipes (*p* < 0.05), while participants in the CIG decreased their opinion about cooking as an interesting activity (*p* < 0.05).

No significant within- or between-group changes related to home cooking habits were observed, including whether participants engaged in cooking (yes or no) and the frequency of cooking in terms of days (≥6 days) and hours (>7 h) per week. Only a significant increase in weekly meal planning was observed among CIG participants (from 39.4% at baseline to 70% after the intervention, *p* = 0.013).

**Figure 3:**
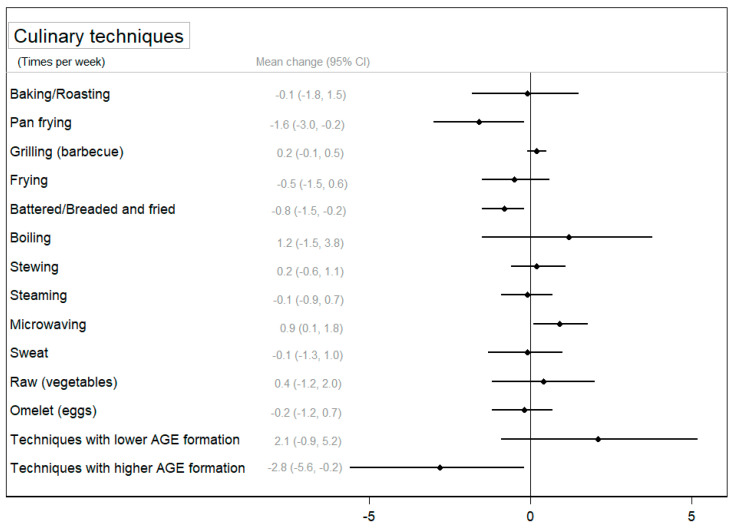
The mean change (95% confidence interval) in the use of cooking techniques between groups after the intervention (change in the CIG–change in the NIG) adjusted for baseline values.

Concerning the use of cooking techniques, Figure 3 and Supplementary Table S6 show the change in the use of cooking techniques between groups after the intervention and its 95% CI. Participants in the CIG significantly decreased the use of pan frying (*p* < 0.05) and battering to frying (*p* < 0.01) after the intervention, and these reductions were larger in the CIG compared to those observed in the NIG (*p* < 0.05). On the contrary, a slight significant increase was observed in the use of microwaving and boiling techniques in the CIG (*p* < 0.05), and for microwaving, this change was statistically significant compared to the NIG. Finally, Figure 3 shows a significant difference between groups in the use of techniques associated with a higher formation of AGEs (*p* < 0.05) after the intervention. Supplementary Table S6 also shows that participants in the CIG significantly reduced the use of techniques associated with a higher formation of AGEs (*p* < 0.01) and improved the use of techniques associated with a lower formation of AGEs (*p* < 0.05).

**Figure 4:**
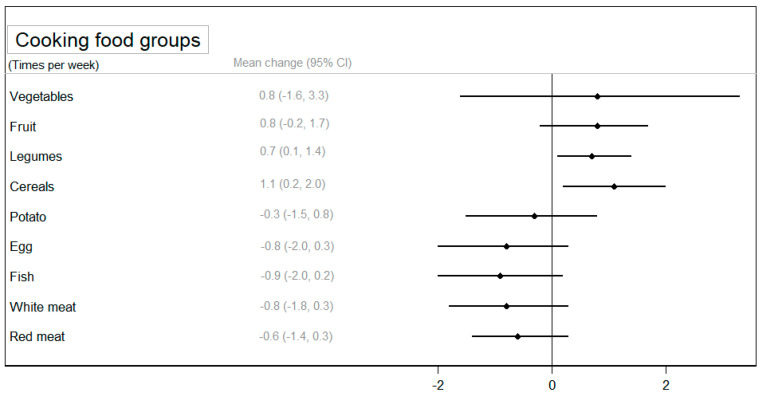
The mean change (95% confidence interval) in the use of food groups for cooking between groups after the intervention adjusted for baseline values.

Regarding the frequency of cooking specific foods, a statistically significant increase was observed in the cooking of legumes (*p* < 0.05) and cereals (*p* < 0.05) in the CIG compared to the NIG (Figure 4). A larger reduction in cooking animal foods (eggs, fish, and red meat) was observed among participants in the CIG, although these differences were not statistically significantly compared to the NIG (Supplementary Table S6).

### 3.3. Health and Biological Outcomes

Table 4 shows the results of the intervention on anthropometric and blood pressure measurements. In terms of within-group changes, a larger and significant reduction in weight (*p* < 0.01), BMI (*p* < 0.01), waist circumference (*p* < 0.05), and hip circumference (*p* < 0.01) was observed in the CIG. The differences in changes between groups were significant for these anthropometric measures, with larger reductions in the CIG compared to the NIG. On the contrary, a larger increase was found in the NIG for fat mass in kilograms (*p* < 0.01) and percentage (*p* < 0.01) and reduced fat-free mass in kilograms (*p* < 0.05) and percentage (*p* < 0.01). Differences in fat mass or fat-free mass changes between groups were not statistically significant. Finally, larger reductions in SBP and DBP were observed within CIG participants, although the difference with the reductions observed in the NIG were not significant.

The effect of the intervention on biochemical determinations is shown in Table 5. A larger and significant reduction in glucose levels was observed in the CIG, although the differences in changes between groups were not statistically significant. Significant increases in LDL-c/HDL-c and TNF-*α* were found within both NIG and CIG participants but no significant differences between groups. No significant differences were found in AGE measurements either between groups or in changes within groups (Supplementary Table S7).

## 4. Discussion

The SUKALMENA-InAge study aimed to evaluate the effectiveness of an intensive culinary intervention programme, as a healthy ageing strategy that may improve healthy eating habits in middle-aged adults with overweight or obesity. This culinary intervention was compared with a more traditional nutritional education programme focused on increasing the adherence to the MedDiet. After a 4-week intervention, participants in the CIG increased their MedDiet adherence more than those assigned to the NIG, mainly due to the increased consumption of legumes, nuts, and vegetables and the reduced consumption of unhealthy fats and sweet beverages in the CIG. Participants in the CIG increased the cooking of plant-based foods and reduced cooking foods from animals. An increase in the overall confidence in cooking was observed in the CIG, as well as a reduction in cooking techniques related to AGE production, with a significant difference compared to the NIG. Furthermore, CIG participants demonstrated improvements in anthropometric measures, including weight, BMI, and waist and hip circumference, compared to the NIG. However, no improvement was observed in overall cooking attitudes or changes in biochemical and AGE measures.

The improvement in MedDiet adherence observed in the CIG compared to the NIG suggests that the development of culinary habits may add additional benefits to improving the knowledge on healthy diets. The MEDAS at baseline was slightly higher in the NIG than in the CIG (8.3 vs. 7.6), but the mixed-effects model analysis allowed for the control of these baseline values. These results confirm that the lack of cooking knowledge and skills is a barrier to dietary modifications [33], and dietary changes toward healthier eating may be facilitated by the acquisition of greater confidence in cooking [34,35]. However, we have to be cautious with this interpretation considering the small sample size. In addition, probably because of the small sample size, only positive trends were observed in the CIG for changes in the food groups used for cooking except for cereals and legumes.

The duration of the culinary intervention was only 4 weeks. This duration was shorter than an intervention previously conducted with participants with diabetes in the SUKALMENA study [17]. The aim was to assess if a more intensive but less time- and resource-consuming intervention could be effective to improve dietary habits in healthy participants. We observed that the culinary intervention significantly increased participants’ overall confidence in cooking, which was translated into specific actions such as greater confidence in preparing balanced meals, modifying recipes, and using leftovers, among others. Moreover, the CIG facilitated the utilization of healthier cooking techniques concurrently with a reduction in the prevalence of less healthy cooking techniques. However, the culinary intervention did not result in better attitudes towards cooking, contrary to previous findings [36]. These findings suggest that there is a need for more larger and personalized interventions to improve confidence in home cooking as well as attitudes towards cooking at home, including identifying and addressing barriers to change, such as the resources needed in the kitchen, previous long-term habits, and cultural issues.

Our results highlight the importance of considering the wider socio-economic and cultural context of home cooking when designing interventions. This is particularly important for healthy people, who are often less motivated to change their behaviour than people with a diagnosed condition. This includes factors such as close personal relationships and household composition, as well as individual considerations such as participants’ time availability and motivation to cook [5]. It is also important to highlight the gender and cultural barriers that should be assessed in a culinary intervention. Globally, women are known to cook more than men [37]. In our study, we observed an imbalance by the sex of the participants between groups, with a higher percentage of women in the CIG. However, we did not observe gender differences in global confidence and attitudes towards cooking. In fact, the between-group differences in MedDiet adherence and other nutritional and cooking-related measures did not change when we additionally adjusted for sex. This lack of differences between women and men can be explained by the fact that the men who volunteered to participate in our study are not representative of the general population, who generally have lower confidence and attitudes towards cooking compared to women. Another interesting point is that the population of the Basque Country is not geographically part of the Mediterranean area, and they may have different food and cooking traditions [38]. It is also a challenge that adherence to the MedDiet is decreasing in the Spanish population as a whole [39,40]. Further research is needed to include men with lower cooking skills and to assess the role that culinary traditions may have as barriers or facilitators in promoting healthy eating and cooking in the context of the MedDiet.

The beneficial outcomes observed in dietary and cooking behaviours in the CIG were translated into significant changes in body composition parameters compared to the NIG and blood pressure measures. The positive effect of the MedDiet on body composition and blood pressure levels has been previously described in the literature [41,42]; however, the evidence regarding the impact of cooking interventions on health parameters such as body composition or blood pressure is scarce, with some authors reporting no effect [36] and other authors demonstrating a positive effect [43,44]. It is noteworthy that although this study was conducted in overweight and obese adults, participants were not counselled to cut back on calories or weight. In this sense, although there are a high number of dietary interventions that promote calorie restriction as the main dietary strategy for achieving healthy ageing [2], as it was previously reported [17], the SUKALMENA-InAge strategy might be beneficial for improving body composition without focusing on calorie restriction. An improvement in food quality and cooking techniques within the CIG could explain this [45,46], as previously reported by Gatto and colleagues [47] who found a significant reduction in BMI and waist circumference and an increased fibre intake after a cooking intervention without reducing energy intake, although this programme was conducted in children. Further research in larger studies is needed to explore the underlying mechanisms associated with this improvement in body composition.

Our study showed no significant differences in biochemical parameters between the groups, although the CIG participants showed a significant increase in TNF-α levels and the LDL-c/HDL-c ratio after the intervention, while glucose levels declined. Overall, the use of biochemical measures in cooking interventions carried out to date appears to be underdeveloped compared to dietary or psychosocial outcomes [48]. Previous findings pointed out the fact that cooking interventions are not associated with significant changes in cardiometabolic risk factors such as LDL-c [36]. However, two previously published studies [49,50] reported significant differences in total cholesterol and the prevalence of hypercholesterolaemia in men after a culinary intervention, although both trials lasted more than five months. Murimi and colleagues [51] pointed out that the intervention duration was an important factor associated with the effectiveness of dietary interventions. Therefore, the effect of culinary interventions on biochemical parameters might be influenced by the duration of the intervention or health profile of the participants. When comparing the results of the present study with those of the previous SUKALMENA study [17], some differences in biochemical parameters (CRP and cholesterol measurements, especially HDL-c) were noted, probably due to the fact that the SUKALMENA study was conducted with patients with type 2 diabetes. In addition, the SUKALMENA study measured three-month changes, whereas the SUKALMENA-InAge study measured only the one-month effect of the intervention. More efforts are needed to define the best design for culinary interventions in order to assess the effect on biochemical measurements in comparison with nutritional interventions [8,48,52].

In our study, we found that CIG participants improved the use of cooking techniques associated with lower AGE formation, such as microwaving or boiling, while techniques exhibiting an elevated formation of AGEs declined. However, although both healthy eating and cooking habits were improved, this was not translated into a reduction in AGE accumulation in the body. Our results contrast with findings from previous studies focused on the promotion on low-AGE diets. Lopez-Moreno and colleagues [53] showed that a low-AGE diet high in MUFAs reduced serum AGE levels, determined by ELISA, after a 12-week trial in seventy-five patients with metabolic syndrome. These authors also conducted a 4-week randomized controlled crossover trial in twenty older adults which showed a significant reduction in postprandial serum AGE levels in the MedDiet group [54]. Vlassara and colleagues [55] carried out a one-year study in obese adults with metabolic syndrome and found a reduction in serum and urine AGE levels in the low-AGE diet group who received cooking training to reduce AGE formation during cooking. Lotan and colleagues [56] conducted a study in older adults and found that an AGE-lowering diet led to a reduction in serum AGEs after 6 months of intervention, which included advice on reducing AGE formation during cooking processes. There are several possible explanations for our negative findings, ranging from the health status of the participants to the design of the intervention [16] and the methods used to measure body AGEs [12]. The small sample size and short duration of the intervention may not be enough to achieve significant results in AGEs assessed using skin fluorescence since it represents the “long-term memory” of accumulated stress [12]. However, we also did not observe any changes in CML measured by immunoassay in serum or urine samples. A previous 4-week crossover randomized trial with 49 participants observed a significant increase in carboxyethyl-lysine associated with a diet high in red and processed meat, but no difference was found for CML, both measured by liquid chromatography/mass spectrometry [57]. These authors found no differences in a similar study in which AGEs were measured by immunoassay [58]. These results could firstly be explained by the high amount of red and processed meat in the experimental group, since carboxyethyl-lysine is more abundant in high-fat meats. In addition, tandem mass spectrometry coupled with liquid chromatography provides higher sensitivity and specificity than ELISA [59]. Therefore, further studies are needed to clarify the relationship between cooking techniques that reduce AGE formation and the total AGE content of the diet and the effect of these changes on body AGE levels. In particular, longer-term interventions that focus more on promoting cooking techniques associated with low AGE production are needed to assess changes in body AGEs from skin, blood, and urine [60]. In addition, chromatographic techniques coupled with mass spectrometry may be a better approach to assess changes in a wide range of AGEs, rather than using fluorometric assays or ELISA.

## 5. Strengths and Limitations

Several shortcomings of the SUKALMENA-InAge study should be mentioned. First, the duration of the intervention was only four weeks, and no longer post-intervention follow-up was measured. Therefore, a longer follow-up will probably be needed to observe changes in attitudes towards cooking as well as AGE reduction as a consequence of cooking-related behaviour changes. Second, the small sample size limits the possibility to conduct stratified analyses in order to assess the potential modification effect of key variables such as gender or cooking attitudes at baseline. Third, the FFQ is a semiquantitative questionnaire, and it is less reliable to measure individual changes in food and nutrient intake [61]. Four, it is recommended that further consideration be given to the determinants of home cooking, with a particular focus on those factors related to personal and socio-cultural influences. Additional objective measures, such as pictures of dishes being cooked at home or new utensils being used in the kitchen, will provide additional information about changes in cooking behaviour beyond the use of questionnaires. Five, the results cannot be extrapolated to participants with some of the exclusion criteria defined in our study, i.e., low motivation to change dietary habits, food allergies or intolerances, or a life-limiting illness or chronic alcoholism. Potential participants with low motivation to change were excluded because it is challenging to achieve relevant changes in habits in a relatively short period of time. Therefore, participants with some of the exclusion criteria could potentially benefit if appropriate cooking interventions are developed in future trials.

Despite these constraints, as strengths of our study, the SUKALMENA-InAge study has a randomized controlled trial design, and this allowed for better control for confounding factors. In addition, a multidisciplinary team consisting of chefs, dieticians, and epidemiologists carried out the methodological design of the culinary intervention, and we used questionnaires specifically designed to measure cooking-related habits. The implementation of classes online permitted the inclusion of a greater range of participants, as it facilitated the participation of individuals who would otherwise not have been able to do so. Finally, the MedDiet was selected as the primary outcome of this study, and, instead of calorie restriction, it was focused on the promotion of food quality and enhancing cooking skills. This strategy could be useful for the promotion of healthy habits in the long term.

## 6. Conclusions

The SUKALMENA-InAge study suggests that an intensive culinary intervention can effectively promote the MedDiet and reduce anthropometric measures compared to a nutritional intervention. The observed increases in the overall confidence in home cooking and the frequency of cooking plant-based foods support the hypothesis that enhancing culinary knowledge and skills may yield additional benefits beyond mere knowledge acquisition regarding healthy eating. However, no significant changes were observed concerning attitudes towards home cooking and its frequency. This suggests the imperative of further exploration into the barriers hindering home cooking and potential solutions. Further studies with larger sample sizes and longer follow-ups are warranted to support our results and elucidate the underlying mechanisms.

## Figures and Tables

**Table 1:** The weekly planning of the culinary intervention programme in the SUKALMENA-InAge study.

Week		1	2	3	4
**Classes**		Mediterranean diet and healthy pantry	Gastro-healthy eating plate	Culinary techniques and AGEs	Food groups: vegetables and fruits, protein, and carbohydrates
		Pantry and shopping	Proportions of plate	Prevention of cooking formation of AGEs	Key aspects of each food group
**Objectives**		Learning organizational and planning guidelines in kitchen	Getting to know proportions of different food groups that healthy dishes should have based on “the healthy eating plate”	Getting to know AGEs and strategies to reduce these compounds in foods	Learning about different proportions of healthy eating plate
**Theoretical part**	**Gastronomic culture**	Mediterranean diet and its benefits	Healthy eating plate	General information about AGEs, what they are, how they are formed, and their effect on health	Vegetables and fruits: benefits, bioactive compounds, and seasonality
		Planning grocery shopping and its key aspects, such as how to read nutritional labels	Extra virgin olive oil: benefits and different types		Proteins: types and plant and animal protein sources
					Carbohydrates: types, food sources, and how to identify whole foods
	**Culinary resources**	Basic equipment	Gastro-healthy eating plate	Strategies to reduce AGEs in foods	Culinary resources to prepare delicious vegetables and fruits
		Pantry: general preparations that boost flavour of plates	Healthy culinary techniques: blanching, boiling, poaching, sweating, grinding, pressure cooking, stewing, use of oven	Healthy culinary techniques: microwaving, papillote, steaming, salt cooking, marinating, pickling, fermenting, sprouting	Culinary resources for preparing delicious dishes based on animal and plant proteins
		Healthy culinary techniques: importance of different cuts	Key culinary strategies: batch cooking		Culinary resources for preparing delicious carbohydrate-based dishes
		How to use recipe books			
**Cooking class**	**Aims**	Preparation of sauces and pastas	Example of healthy eating plate and healthy dessert	Example of how to cook food using different culinary techniques to reduce formation of AGEs	Example of healthy eating plate with vegetal protein and animal protein. Marinating foods in acidic or citrus-based sauces
	**Recipes**	Pesto Green curry Baba ganoush Harissa pasta Asian dressing	Vegetable salmorejo and wholegrain croutons. Ceviche of fruits.	Fish (mackerel) cooked using four culinary techniques: papillote, steaming, salt cooking, and marinating before microwaving	Seasoned boiled leeks, textured soy with garlic and quinoa. Carrots and marinated chicken in papillote with pesto and millet.

AGEs, advanced glycation end products.

**Table 2:** Confidence in cooking at home at baseline and after the 4-week intervention according to the intervention groups in the SUKALMENA-InAge study.

	Within-Group Changes (95% CI)		Difference in Changes between Groups (95% CI)
	NIG (*n* = 26)	CIG (*n* = 30)	
Global confidence about cooking at home ^a^	5.5 (−1.4 to 12.4)	8.3 (2.8 to 13.7) **	2.8 (−6.1 to 11.5)
**Confidence in the use of specific cooking techniques and skills ^a^**			
Prepare food for cooking by chopping, mixing, and stirring	−0.2 (−0.9 to 0.5)	−0.1 (−1.1 to 0.9)	0.1 (−1.1 to 1.3)
Use different methods to cook foods such as boiling	−0.1 (−0.4 to 0.3)	0.3 (−0.7 to 1.2)	0.3 (−0.7 to 1.3)
Use different equipment for cooking	0.02 (−0.5 to 0.5)	0.3 (−0.3 to 1.0)	0.3 (−0.5 to 1.1)
Preserve food	0.1 (−0.9 to 1.0)	1.2 (0.1 to 2.3) *	1.1 (−0.4 to 2.5)
Know when your food is cooked	0.6 (−0.3 to 1.5)	0.6 (−0.04 to 1.2)	−0.01 (−1.1 to 1.1)
Handle, store, and prepare foods safely ^b^	0.1 (−0.6 to 0.7)	−0.2 (−1.0 to 0.5)	−0.3 (−1.3 to 0.7)
Cook grains, for example rice, pasta, etc.	0.2 (−0.4 to 0.7)	0.3 (−0.4 to 1.0)	0.1 (−0.7 to 1.0)
Cook vegetables	0.1 (−0.3 to 0.4)	0.3 (−0.2 to 0.8)	0.3 (−0.3 to 0.8)
Cook meat, fish, or poultry	−0.1 (−0.6 to 0.6)	0.1 (−0.4 to 0.5)	0.1 (−0.6 to 0.8)
Prepare a balanced meal	0.3 (−0.5 to 1.0)	0.8 (0.3 to 1.3) **	0.5 (−0.4 to 1.4)
Prepare more than one food item for a meal at the same time	0.4 (−0.1 to 1.0)	0.5 (−0.1 to 1.1)	0.1 (−0.7 to 0.9)
Compare food prices to save money ^b^	0.4 (−0.1 to 1.6)	0.8 (0.01 to 1.6) *	0.4 (−1.1 to 1.8)
Read the nutrition information on food labels	0.3 (−0.4 to 1.0)	0.5 (−0.2 to 1.2)	0.2 (−0.8 to 1.2)
Plan meals for the week ^c^	0.3 (−0.6 to 1.2)	1.5 (0.5 to 2.5) **	1.2 (−0.2 to 2.5)
Read recipes	−0.5 (−1.2 to 0.3)	0.4 (−0.1 to 1.0)	0.9 (−0.03 to 1.8)
Use substitutions in recipes if I do not have a specific ingredient	0.5 (−0.5 to 1.5)	0.8 (0.1 to 1.4) *	0.3 (−0.9 to 1.4)
Change recipes to make them healthier	1.0 (0.1 to 1.8) *	0.7 (0.2 to 1.2) **	−0.3 (−1.3 to 0.7)
Use leftovers to create another meal ^b^	0.3 (−0.5 to 1.1)	0.7 (0.04 to 1.3) *	0.3 (−0.7 to 1.3)

CI, confidence interval; CIG, culinary intervention group; NIG, nutritional intervention group. ^a^ Values on a scale of 0–180 for the global and 0–10 for each individual item. ^b^ In the CIG, *n* = 29. ^c^ In the NIG, *n* = 25. * *p* < 0.05, ** *p* < 0.01.

**Table 3:** Attitudes about cooking at home at baseline and after the 4-week intervention according to the intervention groups in the SUKALMENA-InAge study.

	Within-Group Changes (95% CI)		Difference in Changes between Groups (95% CI)
	NIG (*n* = 26)	CIG (*n* = 30)	
Global attitude about cooking at home ^a^	1.3 (−5.7 to 8.3)	−1.8 (−11.3 to 7.6)	−3.2 (−14.9 to 8.6)
**Attitude in the use of specific cooking techniques and skills ^a^**			
I do NOT like to cook because it takes too much time.	0.5 (−0.2 to 1.3)	0.5 (−0.4 to 1.4)	−0.03 (−1.2 to 1.1)
Preparing meals at home would NOT improve my health.	0.3 (−1.2 to 1.7)	−0.01 (−0.9 to 0.9)	−0.3 (−2.0 to 1.5)
Cooking meals is a good use of my time ^b^.	−0.5 (−1.5 to 0.4)	0.3 (−0.6 to 1.2)	0.9 (−0.4 to 2.1)
I enjoy cooking.	0.3 (−0.4 to 0.9)	−0.002 (−0.8 to 0.8)	−0.2 (−1.3 to 0.8)
It is important to know how to prepare food.	0.2 (−0.5 to 0.9)	−0.1 (−1.0 to 0.9)	−0.3 (−1.5 to 0.9)
Cooking is fun.	0.02 (−0.7 to 0.7)	0.02 (−0.8 to 0.9)	0.003 (−1.1 to 1.1)
I do NOT like to prepare meals at home because it costs too much money.	0.4 (−0.7 to 1.4)	−0.7 (−1.4 to 0.04)	−1.0 (−2.3 to 0.2)
It is NOT important that I know how to cook.	0.7 (−0.6 to 2.0)	−0.1 (−0.8 to 0.6)	−0.8 (−2.2 to 0.6)
Cooking is interesting.	−0.3 (−1.2 to 0.7)	−0.9 (−1.8 to −0.1) *	−0.7 (−1.9 to 0.6)
Meals made at home are affordable.	0.3 (−0.7 to 1.2)	−0.5 (−1.2 to 0.3)	−0.7 (−1.9 to 0.5)
It is important to eat the recommended 3 portions of fruit each day.	−0.1 (−0.7 to 0.5)	−0.1 (−1.2 to 0.9)	−0.03 (−1.2 to 1.2)
It is important to eat the recommended 2 portions of vegetables each day.	0.3 (−0.3 to 1.0)	0.5 (−0.4 to 1.4)	0.2 (−0.9 to 1.3)
It is easy to prepare meals.	0.4 (−0.3 to 1.1)	−0.4 (−1.1 to 0.4)	−0.8 (−1.8 to 0.2)
Cooking is frustrating.	−0.2 (−1.1 to 0.7)	−0.4 (−1.2 to 0.4)	−0.2 (−1.3 to 1.0)
I like trying new recipes.	−0.8 (−1.6 to −0.1) *	−0.3 (−1.0 to 0.5)	0.6 (−0.5 to 1.6)
It is too much work to cook.	0.2 (−0.8 to 1.2)	−0.1 (−1.2 to 0.9)	−0.3 (−1.7 to 1.1)
Making meals at home helps me to eat more healthily.	0.03 (−0.6 to 0.7)	0.5 (−0.4 to 1.4)	0.5 (−0.6 to 1.5)
I find cooking tiring.	−0.2 (−1.0 to 0.5)	−0.6 (−1.3 to 0.1)	−0.4 (−1.4 to 0.7)

CI, confidence interval; CIG, culinary intervention group; NIG, nutritional intervention group. ^a^ Values on a scale of 0–180 for the global and 0–10 for each individual item. ^b^ In the CIG, *n* = 29. * *p* < 0.05.

**Table 4:** Anthropometric and blood pressure measures at baseline and 4-week changes according to the intervention groups in the SUKALMENA-InAge study.

	Within-Group Changes (95% CI)		Difference in Changes between Groups (95% CI)
	NIG (*n* = 25)	CIG (*n* = 31)	
Weight (kg)	−0.2 (−0.7 to 0.3)	−1.7 (−2.6 to −0.9) ***	−1.5 (−2.5 to −0.5) **
BMI (kg/m^2^)	−0.1 (−0.3 to 0.1)	−0.6 (−0.9 to −0.3) ***	−0.5 (−0.8 to −0.2) **
Waist circumference (cm)	−1.0 (−1.8 to −0.3) **	−2.4 (−3.4 to −1.4) ***	−1.4 (−2.6 to −0.2) *
Hip circumference (cm)	−0.6 (−1.2 to 0.1)	−2.0 (−2.8 to −1.2) ***	−1.4 (−2.4 to −0.4) **
Waist/hip ratio	−0.004 (−0.01 to 0.002)	−0.004 (−0.02 to 0.01)	0.0001 (−0.01 to 0.01)
Fat mass (kg)	1.9 (0.6 to 3.2) **	0.2 (−1.2 to 1.6)	−1.7 (−3.6 to 0.3)
Fat mass (%)	2.2 (0.8 to 3.7) **	0.5 (−1.0 to 1.9)	−1.7 (−3.8 to 0.3)
Fat-free mass (kg)	−1.3 (−3.2 to 0.6) *	−1.8 (−3.5 to −0.2) *	−0.5 (−3.1 to 2.0)
Fat-free mass (%)	−2.3 (−3.7 to −0.8) **	0.5 (−2.0 to 3.0)	2.8 (−0.1 to 5.6)
SBP (mmHg) ^a^	−1.2 (−6.7 to 4.3)	−7.3 (−12.5 to −2.2) **	−6.1 (−13.7 to 1.4)
DBP (mmHg) ^a^	−1.8 (−4.2 to 0.6)	−3.8 (−6.4 to −1.3) **	−2.0 (−5.6 to 1.5)

BMI, body mass index; CI, confidence interval; CIG, culinary intervention group; DBP, diastolic blood pressure; NIG, nutritional intervention group; SBP, systolic blood pressure. ^a^ In the NIG, *n* = 24. * *p* < 0.05, ** *p* < 0.01, *** *p* < 0.001.

**Table 5:** Biochemical measures at baseline and 4-week changes according to the intervention groups in the SUKALMENA-InAge study.

	Within-Group Changes (95% CI)		Difference in Changes between Groups (95% CI)
	NIG (*n* = 23)	CIG (*n* = 26)	
Glucose (mg/dL)	−0.4 (−3.0 to 2.2)	−4.4 (−7.8 to −1.0) *	−4.0 (−8.3 to 0.3)
Insulin (mcU/mL)	0.3 (−0.6 to 1.2)	−0.4 (−2.0 to 1.2)	−0.7 (−2.5 to 1.1)
HOMA-IR	0.02 (−0.2 to 0.3)	−0.2 (−0.8 to 0.3)	−0.3 (−0.9 to 0.4)
Total cholesterol (mg/dL)	3.7 (−6.3 to 13.6)	1.7 (−7.2 to 10.5)	−2.0 (−15.3 to 11.3)
HDL-c (mg/dL)	−1.1 (−3.3 to 1.1)	−2.1 (−5.0 to 0.8)	−1.0 (−4.7 to 2.6)
LDL-c (mg/dL)	5.4 (−2.2 to 13.0)	5.0 (−1.9 to 11.8)	−0.4 (−10.6 to 9.8)
TG (mg/dL)	−4.8 (−22.8 to 13.2)	−5.1 (−19.4 to 9.2)	−0.3 (−23.3 to 22.7)
LDL-c/HDL-c	0.1 (0.02 to 0.2) *	0.2 (0.03 to 0.3) *	0.03 (−0.1 to 0.2)
TG/HDL-c	0.01 (−0.4 to 0.4)	−0.04 (−0.4 to 0.3)	−0.1 (−0.6 to 0.4)
CRP (pg/dL)	0.1 (−0.1 to 0.2)	0.1 (−0.1 to 0.2)	−0.002 (−0.2 to 0.2)
TNF-*α* (mg/dL)	6.6 (0.1 to 13.1) *	7.4 (2.3 to 12.5) **	0.8 (−7.5 to 9.1)

CI, confidence intervals; CIG, culinary intervention group; CRP, C-reactive protein; HDL-c, high-density lipoprotein cholesterol; HOMA-IR, homeostatic model assessment—insulin resistance; LDL-c, low-density lipoprotein cholesterol; NIG, nutritional intervention group; TG, triglycerides; TNF-α, tumour necrosis factor-alpha. * *p* < 0.05, ** *p* < 0.01.

## Data Availability

The data presented in this study are available on request from the corresponding author due to privacy.

## Supplementary Materials

The following supporting information can be downloaded at https://www.mdpi.com/article/10.3390/nu16111735/s1, Table S1: Home cooking Quality Index Questionnaire used as a screening tool in the SUKALMENA-InAge study.; Table S2: Baseline characteristics according to the intervention groups in the SUKALMENA-InAge study.; Table S3: Mediterranean diet adherence at baseline and after 4-week intervention in participants of SUKALMENA-InAge study.; Table S4: Energy and nutrient intake at baseline and after the 4-week intervention according to the intervention groups in the SUKALMENA-InAge study.; Table S5: Food consumption at baseline and after the 4-week intervention according to the intervention groups in the SUKALMENA-InAge study.; Table S6: The use of culinary techniques and food to cook at baseline and after the 4-week intervention according to the intervention groups in the SUKALMENA-InAge study.; Table S7: AGE levels at baseline and 4-week changes according to the intervention groups in the SUKALMENA-InAge study.
