# Supplementary material for: An Intensive Culinary Intervention Programme to Promote Healthy Ageing: The SUKALMENA-InAge Feasibility Pilot Study
Source: Nutrients. 2024 Jun 1;16(11):1735. doi: 10.3390/nu16111735 (PMC11174777; doi:10.3390/nu16111735)
Supplement: Supplementary file 1 [file nutrients-16-01735-s001.zip › nutrients-2995354-supplementary.pdf]

SUPPLEMENTARY TABLES

Table S1. Home cooking Quality Index Questionnaire used as a screening tool in the SUKALMENA-InAge study.

HOMECOOKING QUALITY INDEX	YES	NO
1. Do you eat home-cooked food at least 10 times a week (lunch or dinner)?	1	0
2. Do you eat food prepared at home using techniques such as steaming or papillote at least once a week?	1	0
3. Do you eat vegetables prepared at home using techniques such as poaching, stewing or boiling?	1	0
4. Do you eat meat or fish prepared at home using techniques such as stewing, boiling or pan-frying?	1	0
5. When baking, frying or pan-frying, do you usually leave the food toasted?	0	1
6. Do you eat fried foods 2 times or less per week?	1	0
7. Do you eat food prepared at home with techniques such as grilling or roasting less than once a week?	1	0
8. Do you marinate meat or fish at least once a week?	1	0
9. Do you prefer to cook with extra virgin olive oil?	1	0
10. Do you plan weekly lunches and/or dinners at home?	1	0
11. Do you preferably consume seasonal and/or local food?	1	0
12. Do you include nuts or seeds in your food preparations (salads/sauces) at least once a month?	1	0
13. Do you make homemade pastries with olive oil, wholemeal flour or fruit?	1	0
14. Do you use dried fruit as a sugar substitute in your home baking?	1	0
15. Do you use whole grains (bread/pasta/rice/flour) rather than refined grains (bread/pasta/rice/flour)?	1	0
16. Do you use vegetables instead of processed meats (sausages, black pudding, bacon) when preparing legumes?	1	0
17. Do you practice batch cooking (cooking on one day for several days of the week)?	1	0
18. Do you prepare dishes without salt at least once a day?	1	0
19. Do you use spices at least once a day?	1	0
Summary		
Total score		

Scoring: 0-19 points.
< 10 points: candidate for study
≥ 10 points: NO candidate

Table S2. Baseline characteristics according to the intervention groups in the SUKALMENA-InAge study.

		Mean (SD)		*P* value
		NIG	CIG	
n		29	33	
Sex, n (%)[a]				0.284
Women		11 (37.9)	17 (51.5)	
Men		18 (62.1)	16 (48.5)	
Age (years)[b]		60.7 (4.8)	61.64 (4.6)	0.799
Civil status, married n (%)[a, c, d]		20 (74.1)	27 (90.0)	0.114
Education, n (%)[a, c, d]				0.536
No university		14 (51.9)	18 (60.0)	
University		13 (48.2)	12 (40.0)	
Working status, n (%) [c, d, e]				0.418
Working		14 (51.9)	11 (36.7)	
Retired		12 (44.4)	15 (50.0)	
Other		1 (3.7)	4 (13.3)	
Smoking status, n (%)[a, c, d]				0.695
Never		10 (37.0)	12 (40.0)	
Former		15 (55.6)	14 (46.7)	
Current		2 (7.4)	4 (13.3)	
Mediterranean Diet Adherence Screener (MEDAS). Total score (0-14) [b, d, f]		8.3 (2.4)	7.6 (2.2)	0.454
Score in each MEDAS items (%) [a, d, f]	Criterion			
Do you use olive oil as main cooking fat?	Yes	89.7	97.0	0.242
How much olive oil do you consume in a day (including oil for frying, on salads, between others)?	>4 tablespoon	79.3	84.9	0.569
How many servings of vegetables do you eat per day? (1 serving = 200g)	≥2	27.6	27.3	0.978
How many fruit portions do you eat per day?	≥3	35.7	24.2	0.328
How many servings of red meat, hamburger, or processed meat do you eat per day? (1 portion = 100-150g)	<1	65.5	81.8	0.143

How many servings of butter, margarine, or cream do you eat per day? (1 serving = 12g)	<1	96.6	87.9	0.211
How many sweet beverages do you drink per day? (1 serving = 12g)	<1	89.7	87.9	0.825
How much wine do you drink per week? (glasses)	≥7	27.6	24.2	0.764
How many servings of legumes do you eat per week? (1 portion = 150g)	≥3	31.0	3.0	0.003
How many servings of fish or shellfish do you eat per week? (1 portion = 150/200g)	≥3	58.6	48.5	0.425
How many times per week do you eat commercial sweets or pastries (not homemade)?	<2	61.5	51.5	0.441
How many servings of nuts do you eat per week? (1 potion = 30g)	≥3	41.4	39.4	0.874
Do you preferably eat chicken, turkey, or rabbit meat instead of beef, pork, hamburger, or sausage?	Yes	62.1	66.7	0.706
How many times per week do you eat vegetables, pasta, rice or other dishes seasoned with tomato, garlic, onion or leek sauce cooked in olive oil (sauté)?	>2	72.4	33.3	0.002
Weight (kg) [b, h, i]		87.3 (12.5)	85.8 (15.2)	0.429
BMI (kg/m^2) [g, h, i]		30.5 (3.1)	29.8 (3.2)	0.165
Waist (cm) [b, h, i]		106.6 (10.3)	106.0 (8.1)	0.441
Hip (cm) [g, h, i]		109.4 (8.9)	107.9 (7.9)	0.778
Waist/hip ratio [b, h, i]		1.0 (0.1)	1.0 (0.1)	0.697
Fat mass (kg) [b, h, i]		25.2 (8.3)	23.4 (7.4)	0.407
Fat mass (%) [b, h, i]		29.0 (9.0)	27.6 (8.8)	0.745
Fat-free mass (kg) [b, h, i]		61.4 (14.0)	62.4 (14.9)	0.888
Fat-free mass (%) [b, h, i]		71.0 (9.0)	72.4 (8.8)	0.742
SBP (mmHg) [b, i, j]		137.3 (17.9)	141.9 (18.8)	0.522
DBP (mmHg) [b, i, j]		85.6 (9.0)	88.6 (10.3)	0.449
Glucose (mg/dL) [g, k, l]		94.6 (8.8)	102.8 (22.3)	0.667
Insulin (mcU/mL) [g, k, l]		9.3 (5.1)	8.6 (4.7)	0.135
HOMA-IR [g, k, l]		2.2 (1.2)	2.3 (1.7)	0.254

Total cholesterol (mg/dL) [b, k, l]	218.9 (44.8)	209.7 (42.4)	0.470
HDLc (mg/dL) [g, k, l]	59.0 (13.3)	58.7 (16.3)	0.847
LDLc (mg/dL) [b, k, l]	136.2 (35.4)	125.7 (34.7)	0.439
TG (mg/dL) [g, k, l]	119.4 (44.8)	126.7 (63.6)	0.192
LDL/HDL [b, k, l]	2.3 (0.6)	2.3 (0.8)	0.533
TG/HDL [g, k, l]	2.1 (1.0)	2.4 (1.6)	0.347
CRP (pg/dL) [g, k, l]	0.3 (0.2)	0.3 (0.3)	0.814
TNF-α (mg/dL) [g, k, l]	9.8 (4.4)	8.2 (4.2)	0.132
**Global confidence about cooking at home** [b, d, f, m]	129.2 (32.3)	131.7 (21.7)	0.799
Prepare food for cooking by chopping, mixing and stirring [d, f, g]	7.5 (2.5)	7.7 (2.0)	0.960
Use different methods to cook foods such as boiling [d, f, g]	7.9 (1.8)	7.5 (2.3)	0.500
Use different equipment for cooking [d, f, g]	8.0 (2.1)	7.7 (1.8)	0.164
Preserve food [d, f, g]	6.5 (2.5)	6.2 (2.9)	0.830
Know when your food is cooked [b, d, f]	6.1 (3.0)	6.7 (2.2)	0.252
Handle, store and prepare foods safely [f, g, n]	6.7 (2.8)	7.9 (1.9)	0.170
Cook grains, for example, rice, pasta, etc [d, f, g]	7.9 (2.1)	7.8 (2.3)	0.874
Cook vegetables [d, f, g]	8.5 (1.5)	8.3 (1.6)	0.799
Cook meat, fish, or poultry [d, f, g]	8.3 (1.7)	7.9 (1.4)	0.316
Prepare a balance meal [b, d, f]	6.6 (2.1)	7.3 (2.0)	0.129
Prepare more than one food item for a meal at the same time [d, f, g]	7.3 (2.7)	7.5 (1.9)	0.954
Compare food prices to save money [f, g, n]	6.0 (3.3)	6.1 (3.3)	0.913
Read the nutrition information on food labels [d, f, g]	5.9 (2.6)	6.8 (2.8)	0.081
Plan meals for the week [b, d, h]	5.1 (2.7)	4.9 (2.9)	0.563
Read recipes [d, f, g]	7.2 (2.6)	7.6 (1.6)	0.903
Use substitutions in recipes if I don't have a specific ingredient [d, f, g]	6.6 (3.1)	7.1 (2.0)	0.694

Change recipes to make them healthier [d, f, g]		6.7 (2.7)	7.3 (2.2)	0.246
Use leftovers to create another meal [f, g, n]		7.0 (2.7)	7.0 (2.1)	0.391
**Global attitude about cooking at home** [d, f, g, m]		142.9 (23.4)	149.0 (13.6)	0.454
I do NOT like to cook because it takes too much time [d, f, g]		7.3 (2.9)	8.2 (2.5)	0.457
Preparing meals at home would NOT improve my health [d, f, g]		8.4 (2.8)	8.8 (2.0)	0.869
Cooking meals is a good use of my time [f, g, n]		7.6 (2.0)	7.5 (2.4)	0.912
I enjoy cooking [d, f, g]		6.9 (2.7)	8.0 (1.8)	0.367
It is important to know how to prepare food [d, f, g]		8.9 (2.2)	8.7 (2.0)	0.595
Cooking is fun [d, f, g]		7.4 (2.2)	7.9 (1.6)	0.596
I do NOT like to prepare meals at home because it costs too much money [d, f, g]		8.4 (2.7)	9.7 (0.6)	0.059
It is NOT important that I know how to cook [d, f, g]		8.3 (2.9)	8.3 (2.7)	0.866
Cooking is interesting [d, f, g]		8.4 (2.0)	8.8 (1.2)	0.386
Meals made at home are affordable [d, f, g]		8.2 (2.5)	8.7 (1.3)	0.493
It is important to eat the recommended 3 portions of fruit each day [d, f, g]		8.5 (1.5)	7.8 (2.2)	0.365
It is important to eat the recommended 2 portions of vegetables each day [d, f, g]		8.3 (1.7)	7.7 (2.0)	0.234
It is easy to prepare meals [d, f, g]		7.3 (2.5)	8.4 (1.3)	0.178
Cooking is frustrating [d, f, g]		8.7 (1.7)	9.0 (1.5)	0.552
I like trying new recipes [d, f, g]		8.0 (2.1)	8.0 (2.0)	0.822
It is too much work to cook [b, d, f]		5.7 (2.6)	6.5 (2.4)	0.174
Making meals at home helps me to eat more healthfully [d, f, g]		8.6 (1.7)	8.4 (1.7)	0.810
I find cooking tiring [d, f, g]		8.4 (2.0)	8.9 (0.9)	0.837
Weekly meal planning, n (%)[a, d, f]	Planification	12 (41.4)	13 (39.4)	0.874
In charge of weekly grocery shopping, n (%)[a, d, f]	Yes	22 (75.9)	33 (100.0)	0.003
Cook, n (%)[a, d, f]	Yes	28 (96.6)	32 (97.0)	0.926
Cook days per week n (%)[a, d, f]	≥6 days	16 (57.1)	22 (68.8)	0.352

Cook hours per week, n (%)[a, d, f]	>7 hours	13 (46.4)	22 (68.8)	0.080
Use of culinary techniques (times per week)				
Baking/Roasting [d, f, g]		2.9 (1.9)	2.8 (2.9)	0.697
Pan frying [d, f, g]		3.9 (2.8)	4.2 (3.2)	0.713
Grilling (barbecue) [d, f, g]		0.3 (0.7)	0.1 (0.4)	0.181
Frying [d, f, g]		2.9 (1.8)	2.3 (3.0)	0.133
Battered/Breaded and fried [d, f, g]		0.7 (0.8)	1.2 (1.7)	0.310
Boiling [d, f, g]		10.4 (4.3)	7.7 (3.3)	0.009
Stewing [d, f, g]		0.7 (1.0)	0.9 (1.2)	0.604
Steaming [d, f, g]		0.4 (1.0)	0.8 (1.5)	0.687
Microwaving [d, f, g]		0.4 (0.8)	0.5 (1.4)	0.430
Sweat [d, f, g]		1.2 (2.0)	1.1 (1.4)	0.364
Raw (vegetables) [d, f, g]		3.9 (2.4)	4.2 (2.6)	0.875
Omelet (eggs) [d, f, g]		2.1 (1.4)	1.8 (1.2)	0.442
Use of food to cook (times per week)				
Vegetables [d, f, g]		9.2 (3.9)	9.2 (4.6)	0.667
Fruit [d, f, g]		0.5 (1.4)	0.1 (0.3)	0.069
Legumes [b, d, f]		1.9 (1.3)	1.4 (0.8)	0.095
Cereals [d, f, g]		2.0 (1.4)	1.5 (0.9)	0.302
Potato [d, f, g]		3.2 (1.5)	2.5 (2.0)	0.074
Egg [d, f, g]		4.4 (2.0)	4.0 (1.9)	0.369
Fish [b, d, f]		3.9 (1.8)	3.6 (1.5)	0.824
White meat [b, d, f]		2.8 (1.2)	3.2 (2.0)	0.649
Red meat [d, f, g]		2.3 (1.5)	2.1 (1.6)	0.560

BMI, Body mass index; CIG, Culinary intervention group; CRP, C reactive protein; DBP, Diastolic blood pressure; HDLc, High density lipoprotein cholesterol; HOMA-IR, Homeostatic model assessment-insulin resistance; LDLc, Low density lipoprotein cholesterol; NIG, Nutritional intervention group; SBP, Systolic blood pressure; SD, Standard deviation; TG, Triglycerides; TNF-α, Tumor necrosis factor-alpha.

[a] Difference in baseline between groups for categorical variables. χ^2.
[b] Difference in baseline between groups for continuous variables with normal distribution. T-student test.
[c] In the NIG n=27.
[d] In the CIG n=30.
[e] Baseline difference between groups for categorical variables if one category < 5%. Fisher's exact test.
[f] In the NIG n=26.
[g] Difference in baseline between groups for continuous variables without normal distribution. Mann-Whitney *U* test.
[h] In the NIG n=25.
[i] In the CIG n=31.
[j] In the NIG n=24.
[k] In the NIG n=23.
[l] In the CIG n=26.
[m] Values on a scale of 0-180 for the global and of 0-10 for each individual item.
[n] In the CIG n=29.

Table S3. Mediterranean diet adherence at baseline and after 4-week intervention in participants of the SUKALMENA-InAge study.

**Mediterranean Diet Adherence Screener (MEDAS) items**		**Within-group changes (95 % CI)**		**Difference of changes between groups (95 % CI)**
		**NIG (n=26)**	**CIG (n=30)**	
Do you use olive oil as main cooking fat?	Yes	5.0 (-2.6 to 12.7)	3.2 (-3.0 to 9.4)	-1.8 (-11.7 to 8.0)
How much olive oil do you consume in a day (including oil for frying, on salads, between others)?	>4 tablespoon	16.5 (2.8 to 30.2)*	5.5 (-9.8 to 20.8)	-11.0 (-31.6 to 9.5)
How many servings of vegetables do you eat per day? (1 serving = 200g)	≥2	13.9 (-10.2 to 38.0)	26.2 (7.9 to 44.6)**	12.3 (-18.0 to 42.6)
How many fruit portions do you eat per day?	≥3	3.5 (-10.0 to 16.9)	8.2 (-8.8 to 25.3)	4.8 (-16.9 to 26.5)
How many servings of red meat, hamburger, or processed meat do you eat per day? (1 portion = 100-150g)	<1	10.9 (-12.5 to 34.4)	10.9 (-2.8 to 24.7)	-0.04 (-27.2 to 27.2)
How many servings of butter, margarine, or cream do you eat per day? (1 serving = 12g)	<1	3.5 (-3.3 to 10.1)	12.1 (0.9 to 23.4)*	8.7 (-4.4 to 21.7)
How many sweet beverages do you drink per day? (1 serving = 12g)	<1	7.0 (-2.8 to 16.7)	9.9 (0.3 to 19.6)*	2.9 (-10.8 to 16.7)
How much wine do you drink per week? (glasses)	≥7	6.5 (-4.0 to 17.0)	0.6 (-12.4 to 13.7)	-5.9 (-22.6 to 10.9)
How many servings of legumes do you eat per week? (1 portion = 150g)	≥3	-7.9 (-28.7 to 12.9)	37.4 (19.0 to 55.8)***	45.3 (17.5 to 73.1)**
How many servings of fish or shellfish do you eat per week? (1 portion = 150/200g)	≥3	-1.6 (-21.6 to 18.3)	-1.4 (-25.5 to 22.8)	0.2 (-31.1 to 31.5)
How many times per week do you eat commercial sweets or pastries (not homemade)?	<2	5.3 (-14.2 to 24.7)	19.0 (-2.2 to 40.2)	13.7 (-15.1 to 42.5)
How many servings of nuts do you eat per week? (1 potion = 30g)	≥3	2.2 (-17.6 to 22.1)	18.9 (0.4 to 37.4)*	16.7 (-10.5 to 43.8)
Do you preferably eat chicken, turkey, or rabbit meat instead of beef, pork, hamburger, or sausage?	Yes	17.7 (2.7 to 32.7)*	15.7 (3.5 to 27.9)*	-2.0 (-21.3 to 17.4)
How many times per week do you eat vegetables, pasta, rice or other dishes seasoned with tomato, garlic, onion or leek sauce cooked in olive oil (*sofrito*)?	>2	-30.1 (-55.4 to -4.8)*	0.0 (0.0 to 0.0)	30.1 (-4.4 to 64.6)

CI, Confidence intervals; CIG, Culinary intervention group; NIG, Nutritional intervention group.
*$p<0{\cdot}05$, **$p<0{\cdot}01$, ***$p<0{\cdot}001$.

Table S4. Energy and nutrient intake at baseline and after 4-week intervention according to the intervention groups in the SUKALMENA-InAge study.

	**Within-group changes (95 % CI)**		**Difference of changes between groups (95 % CI)**
	**NIG (n=12)**	**CIG (n=14)**	
**Total energy, kcal/day**	-325.1 (-588.6 to -61.5)*	-211.2 (-530.5 to 108.2)	113.9 (-300.1 to 528.0)
**Macronutrients**			
Carbohydrate intake, % of TEI	-1.8 (-5.1 to 1.5)	-7.1 (-9.7 to -4.4)***	-5.3 (-9.5 to -1.1)*
Glycemic load	-21.8 (-43.8 to 0.3)	-43.7 (-70.3 to -17.0)**	-21.9 (-56.5 to 12.6)
Glycemic index	0.3 (-2.2 to 2.7)	-5.2 (-8.6 to -1.9)**	-5.5 (-9.7 to -1.3)*
Protein intake, % of TEI	-0.4 (-1.5 to 0.6)	-0.6 (-1.8 to 0.7)	-0.2 (-1.8 to 1.5)
Animal protein, g/day	-12.8 (-24.9 to -0.7)*	-9.3 (-15.5 to -3.0)**	3.5 (-10.1 to 17.2)
Vegetal protein, g/day	-3.4 (-7.2 to 0.5)	-4.3 (-11.0 to 2.4)	-0.9 (-8.7 to 6.8)
Fat intake, % of TEI	2.7 (-0.3 to 5.8)	6.8 (3.7 to 9.8)***	4.1 (-0.3 to 8.4)
SFA intake, % of TEI	-0.3 (-1.6 to 1.1)	0.3 (-0.6 to 1.2)	0.6 (-1.0 to 2.2)
MUFA, % of TEI	2.7 (0.1 to 5.3)*	5.7 (3.1 to 8.2)***	3.0 (-0.7 to 6.6)
PUFA, % of TEI	-0.2 (-0.9 to 0.6)	1.0 (0.3 to 1.7)**	1.2 (0.2 to 2.2)*
Cholesterol, mg/day	-47.3 (-203.4 to 108.7)	-50.0 (-119.7 to 19.7)	-2.6 (-173.5 to 168.3)
Fiber, g/day	-0.8 (-4.2 to 2.6)	-1.2 (-6.4 to 4.0)	-0.4 (-6.7 to 5.8)
Alcohol intake, g/day	-2.5 (-5.6 to 0.5)	3.1 (-6.1 to 12.3)	5.6 (-4.1 to 15.3)
Na, mg/day	-816.1 (-1210.2 to -421.9)***	-552.2 (-1012.1 to -92.3)*	263.9 (-341.9 to 869.6)

CI, Confidence intervals; CIG, Culinary intervention group; MUFA, Monounsaturated fatty acids; NIG, Nutritional intervention group; PUFA, Polyunsaturated fatty acids; SFA, Saturated fatty acids; TEI, Total energy intake.
*p<0·05, **p<0·01, ***p<0·001.

Table S5. Food consumption at baseline and after 4-week intervention according to the intervention groups in the SUKALMENA-InAge study.

	**Within-group changes (95 % CI)**		**Difference of changes between groups (95 % CI)**
	**NIG (n=12)**	**CIG (n=14)**	
Vegetables, g/day	50.8 (-53.9 to 155.6)	20.4 (-41.1 to 82.0)	-30.4 (-151.9 to 91.0)
Fruits, g/day	9.1 (-72.1 to 90.2)	-40.0 (-121.6 to 41.7)	-49.0 (-164.1 to 66.1)
Cereals and grains, g/day	-45.2 (-82.1 to -8.4)*	-65.6 (-131.4 to 0.2)	-20.4 (-95.8 to 55.0)
Potatoes, g/day	-40.3 (-76.5 to -4.2)*	-76.3 (-148.6 to -4.0)*	-36.0 (-116.9 to 44.8)
Legumes, g/week	33.3 (-4.9 to 71.5)	58.1 (-5.6 to 121.7)	24.8 (-49.4 to 99.0)
Nuts, g/week	-15.9 (-72.8 to 41.0)	25.7 (-35.9 to 87.2)	41.5 (-42.3 to 125.4)
Dairy products, g/week	-554.4 (-1325.0 to 216.1)	-337.3 (-878.8 to 204.2)	217.1 (-724.7 to 1158.9)
Meat, g/week	-103.4 (-325.0 to 118.3)	-234.7 (-363.0 to -106.4)***	-131.3 (-387.5 to 124.8)
Lean meat, g/week	61.9 (-49.6 to 173.3)	-5.7 (-95.5 to 84.2)	-67.5 (-210.7 to 75.6)
Red meat, g/week	-172.7 (-341.6 to -3.8)*	-216.8 (-340.0 to -93.6)**	-44.2 (-253.2 to 164.9)
Fish	-94.5 (-240.6 to 51.6)	-51.9 (-222.5 to 118.8)	42.7 (-182.0 to 267.3)
White fish, g/week	-44.6 (-172.3 to 83.2)	-37.5 (-177.8 to 102.8)	7.1 (-182.7 to 196.8)
Blue fish, g/week	-53.2 (-138.5 to 32.1)	-10.3 (-85.3 to 64.8)	42.9 (-70.7 to 156.6)
Factory-baked goods, g/week	-49.0 (-149.5 to 51.4)	-134.9 (-229.3 to -40.5)**	-85.8 (-223.7 to 52.0)
Eggs, g/day	-3.2 (-39.2 to 32.8)	2.0 (-13.5 to 17.5)	5.2 (-34.0 to 44.4)
Olive oil, g/day	1.7 (0.8 to 2.6)***	0.1 (-1.0 to 1.3)	-1.6 (-3.0 to -0.1)*

CI, Confidence intervals; CIG, Culinary intervention group; NIG, Nutritional intervention group.
*p<0·05, **p<0·01, ***p<0·001.

Table S6. The use of culinary techniques and food to cook at baseline and after 4-week intervention according to the intervention groups in the SUKALMENA-InAge study.

Use of culinary techniques (times per week)	Within-group changes (95 % CI)		Difference of changes between groups (95 % CI)
	NIG (n=26)	CIG (n=30)	
Baking/Roasting	-0.2 (-1.0 to 0.7)	-0.3 (-1.7 to 1.1)	-0.1 (-1.8 to 1.5)
Pan frying	0.5 (-0.5 to 1.4)	-1.2 (-2.1 to -0.2)*	-1.6 (-3.0 to -0.2)*
Grilling (barbecue)	-0.2 (-0.4 to 0.04)	0.03 (-0.2 to 0.2)	0.2 (-0.1 to 0.5)
Frying	-1.0 (-1.7 to -0.3)**	-1.5 (-2.2 to -0.7)***	-0.5 (-1.5 to 0.6)
Battered/Breaded and fried	0.04 (-0.4 to 0.4)	-0.8 (-1.4 to -0.3)**	-0.8 (-1.5 to -0.2)*
Boiling	0.6 (-1.4 to 2.6)	1.7 (0.1 to 3.4)*	1.2 (-1.5 to 3.8)
Stewing	-0.2 (-0.6 to 0.2)	0.1 (-0.7 to 0.8)	0.2 (-0.6 to 1.1)
Steaming	0.04 (-0.5 to 0.6)	-0.04 (-0.6 to 0.6)	-0.1 (-0.9 to 0.7)
Microwaving	-0.1 (-0.4 to 0.2)	0.8 (0.02 to 1.6)*	0.9 (0.1 to 1.8)*
Sweat	0.001 (-0.9 to 0.9)	-0.1 (-0.8 to 0.6)	-0.1 (-1.3 to 1.0)
Raw (vegetables)	0.1 (-1.0 to 1.2)	0.5 (-0.7 to 1.6)	0.4 (-1.2 to 2.0)
Omelet (eggs)	-0.1 (-0.9 to 0.7)	-0.3 (-0.8 to 0.1)	-0.2 (-1.2 to 0.7)
**Techniques associated with lower AGE formation**	0.3 (-1.5 to 2.2)	2.4 (0.01 to 4.9)*	2.1 (-0.9 to 5.2)
**Techniques associated with a higher formation of AGE**	-0.8 (-2.4 to 0.8)	-3.7 (-5.8 to -1.5)**	-2.8 (-5.6 to -0.2)*
Vegetables	0.9 (-1.0 to 2.7)	1.7 (0.03 to 3.3)*	0.8 (-1.6 to 3.3)
Fruit	-0.01 (-0.2 to 0.2)	0.7 (-0.2 to 1.7)	0.8 (-0.2 to 1.7)
Legumes	-0.2 (-0.7 to 0.3)	0.5 (0.1 to 1.0)*	0.7 (0.1 to 1.4)*
Cereals	-0.4 (-0.8 to 0.1)	0.7 (-0.1 to 1.5)	1.1 (0.2 to 2.0)*
Potato	-0.1 (-1.1 to 0.8)	-0.4 (-1.1 to 0.2)	-0.3 (-1.5 to 0.8)
Egg	-0.2 (-1.1 to 0.7)	-1.1 (-1.8 to -0.4)**	-0.8 (-2.0 to 0.3)
Fish	-0.2 (-0.7 to 0.3)	-1.1 (-2.0 to -0.2)*	-0.9 (-2.0 to 0.2)
White meat	0.2 (-0.5 to 0.8)	-0.6 (-1.5 to 0.3)	-0.8 (-1.8 to 0.3)
Red meat	-0.6 (-1.1 to -0.02)*	-1.1 (-1.7 to -0.5)***	-0.6 (-1.4 to 0.3)

AGE, Advanced glycation end products; CI, Confidence intervals; CIG, Culinary intervention group; NIG, Nutritional intervention group.

*p<0·05, **p<0·01, ***p<0·001.

Table S7. AGE levels at baseline and 4-week changes according to the intervention groups in the SUKALMENA-InAge study.

	Within-group changes (95 % CI)		Difference of changes between groups (95 % CI)
	**NIG (n=23)**	**CIG (n=26)**	
Urine AGE-CML (ng/mL)	-11.8 (-30.2 to 6.7)	6.8 (-4.5 to 18.2)	18.6 (-3.1 to 40.3)
Serum AGE-CML (ng/mL)	-32.2 (-184.9 to 120.6)	-59.9 (-190.9 to 71.1)	-27.7 (-228.9 to 173.5)
RAGE (pg/mL)	-31.6 (-105.3 to 42.1)	-41.9 (-91.3 to 7.4)	-10.4 (-99.1 to 78.3)
	**NIG (n=25)**	**CNIG (n=31)**	
Skin AGE	0.1 (-0.04 to 0.2)	-0.002 (-0.1 to 0.1)	-0.1 (-0.2 to 0.1)

AGE, Advanced glycation end products; AGE-CML, Advanced glycation end products-carboxy-methyl lysine; CI, Confidence intervals; CIG, Culinary intervention group; NIG, Nutritional intervention group; RAGE, Receptor for advanced glycation end products.
